# *In silico* comparison between the mutated and wild type androgen receptors and their influence on the selection of optimum androgenic receptor blocker for treatment of prostate cancer

**DOI:** 10.12688/f1000research.110072.1

**Published:** 2022-05-12

**Authors:** Hany Akeel Al-Hussaniy, Zahraa S. Al-tameemi, Mohammed J. AL-Zobaidy

**Affiliations:** 1 Department of Pharmacology, College of Anesthetic, Alnukbah University, Baghdad, Iraq; 2 Department of Pharmacology, College of Medicine, University of Baghdad, Baghdad, Iraq; 3 Department of Pharmacognosy and Medicinal Plants, College of Pharmacy, University of Baghdad, Baghdad, Iraq

**Keywords:** androgenic antagonist, anticancer, androgen receptor, molecular docking

## Abstract

**Background**: Prostate cancer is a disease that occurs in men aged more than 50 years. In Iraq, 8.89 men per 100,000 population suffer from prostate cancer, with the incidence being 14,016 cases and mortality being 6,367 cases. Despite advances in treatment against prostate cancer, it can become resistant to drugs. Therefore, the aim of current study was to search and identify binding sites for the repositioning of drugs by computational methods (docking).

**Methods**: Based on the protein structure of the wild androgen receptor, the analysis parameters (22x22x22 on the X, Y, and Z axes) were established.

**Results**: The interactions of the natural ligands with androgen receptor were 10.0 (testosterone) and 10.8 (dihydrotestosterone) while mutated androgen receptor (T877A) had a low affinity with testosterone and dihydrotestosterone (-5.3 and -6.7, respectively). In the interactions of both receptors with the reported inhibitors (antagonists), a decrease with Bicalutamide (-8.3 and -4.3, respectively) and an increase in affinity with Flutamide and Nilutamide (-7.7 and 8.6, wild AR; -8.7 and -9.3 AR T877A) were observed. As for Enzalutamide and Apalutamide (second-generation antagonists), the change was minimal between wild androgen receptor and T877A (-7.6 and -7.7; -7.3 and -7.3, respectively). The change in the affinity of the ligands with androgen receptor and androgen receptor T877A shows how a mutation alters the bonds between these molecules.

**Conclusion**: The identification of key sites and potent inhibitors against abnormal androgen receptor functions will enrich prostate cancer treatments.

## Introduction

Prostate cancer (PCa) is a malignant growth commonly occurs in men over the age of 50 years and consists of an increase in prostate size due to an increase in the number of cells. In Iraq, this disease has low incidence and mortality, according to previous studies, about 7.6% of all types of cancers is prostate cancer. ^1^^,^^2^ In addition, the most likely age for development is between 50 and 74 years. Furthermore, progression of the PCais related to excess androgen stimulation and treatments include surgery and/or hormones that completely block androgenic receptor. ^3^^,^^4^

When the disease reaches a stage of resistance to treatments, it is called Castration-Resistant Prostate Cancer (CRPC). ^5^ Although new treatment strategies have been developed for CRPC, they are very few and quite inefficient. Many types of CRPC rely on decreasing the activity of the androgen receptor (AR) signaling the pathway for survival. Due to this, the androgen receptor is key to the design of new therapeutic strategies. ^5^ To date, more than 600 different mutations have been found in the androgen receptor where the repercussions of these mutations on their structure, signaling, and resistance to PCa treatments are analyzed. For this reason, the development of strategies for identification of effective drugs acting on androgen receptors is of great importance in obtaining new therapeutic agents against PCa. ^6^ This methodology is known as Intensive Structure-Based Virtual Screening (SBVS). ^5^^,^^6^ With the SBVS you can identify new bioactive compounds, make modifications in the structure of molecules, and look for the conformation and optimal position of a ligand with its target molecule so as to adjust it for the purpose of the drug. ^6^^–^^9^ In addition, the methodology of drug repositioning, which accelerates the process of discovering new uses of existing therapeutic agents, allows the cost and time of their development to be significantly reduced. Therefore, the use of computational technologies based on protein structure (SBVS) for the design or repositioning of drugs (new uses of existing drugs) is an alternative method that will favor the investigation of new therapeutic agents against CRPC. ^10^ Therefore, the aim of current study was to find and identify binding sites for both wild type and mutated androgenic receptor and best proposed drug that antagonized mutated one by computational methods (docking).

## Methods

The *in silico* experiments so far were carried out at the Center for Research in Iraqi Medical and Pharmaceutical Research Center (IMRC). Subsequently, background analysis will be carried out with services provided by protein Data bank-EMBL-EBI (The European Bioinformatics Institute). Current work is a preliminary trial for the selection of pharmacological molecules with inhibitory potential of the normal and mutated androgen receptor (T877A). ^11^^–^^19^

### Collection of 3D structures of the wild and mutated androgen receptor (T877A)

This first stage consisted of obtaining 3D structures of the wild androgen receptor (AR) and a mutated one (T877A) which is the mutant associated with drug resistance. The 3D molecules were obtained through the database of the protein data bank with access codes 2AM9 (wild) and 2AX6 (mutation).

### Collection of the 3D structures of natural ligands and androgen receptor inhibitors

The structures of the natural ligands of the receptor (Testosterone and Dihydrotestosterone) and its inhibitors (Bicalutamide, Nilutamide, Flutamide, Enzalutamide, and Apalutamide) were obtained from the free database ZINC ( http://zinc15.docking.org). These ligands were selected based on the affinity reported in the database ChEMBL.

### Adequacy of the wild and mutated androgen receptor

The preparation of AR was carried out through the Chimera USFC program, and this preparation consisted of the addition of hydrogen atoms, removal of water molecules from the protein surface, elimination of ligands present, and the determination of charges that integrate each atom of the receiver. In this case, the selected charges were the AM-BCC. Subsequently, it was identified and selected based on the basis of the basic characteristics of the active androgen receptor site according to PDB data. ^12^

### Adequacy of the natural ligands and androgen receptor inhibitors

Ligands, both natural and those reported as inhibitors of AR activity, were subjected to adaptations with the Chimera USFC program for molecular interaction assays with the in-silico androgen receptor, adding hydrogen atoms and assigning Gastieger charges to mimic the changes that occur within a cell according to their nature. ^13^

### Assays of wild AR interactions with natural ligands and AR inhibitors to establishing interaction parameters

The calculation of the affinity values (Kcal/mol) was performed using the AutoDock program, the interactions between wild AR and its natural ligands were analyzed, and the contact between the molecules when the distance was less than or equal to 5 Å was considered. On the basis of the interaction of natural ligands (Testosterone and Dihydrotestosterone), the optimal parameters for the evaluation of receptor interactions were determined. For the validation of these parameters, the interaction between the wild androgen receptor with the five inhibitors were reported against the receptor (Bicalutamide, Nilutamide, Flutamide, Enzalutamide, and Apalutamide). It is considered the same distance (5 Å) for a true interaction between ligands with AR. In addition, within the validation of the joining site, the size assessment of the coupling site was carried out for the structures of the ligands which was defined by establishing a cube with the dimensions of 22×22×22 Å, 15×15×15Å, and 10×10×10 Å. This was in order to experimentally determine the exact key parameters for the interactions between the receptor and ligands. ^14^

### Assays of interactions of mutated AR (T877A) with natural ligands and AR inhibitors

The interaction analyses between the mutated androgen receptor (T877A) were performed based on the above-mentioned calculations, taking into account the size of the binding site determined for wild AR with natural receptors and inhibitors (22×22×22 Å). This is in order to determine if the mutation changes the affinity between the receptor and the molecules evaluated.

### Identification of the main residues that interact between the wild and mutated AR (T877A) and the ligands

The identification of residues of the AR that interact with the natural ligands and inhibitors that had the best affinity score in the analyses in AutoDock Vina (Kcal/mol) were visualized using the PyMOL program with which each complex between the receptor and the ligand was observed.

## Results

The first result obtained from the analysis was carried out with the help of the optimal parameters of the AR binding site which were determined with the use of AutoDock Vina and Chimera. The parameters are described below ( Table 1).

**Table 1.** Wild androgen receptor binding site parameters (2AM9).

	X	Y	Z
Center	24.23	4.61	6.129
Size	22	22	22

With the server of AutoDock Vina de Chimera and AutoDock Vina (direct program), the calculation of the theoretical value for the affinity of the coupling of the natural ligand and inhibitors with the androgen receptors, both wild and mutated. The following Tables 2 and 3 showed the analyses of affinities existed in the interactions between the wild AR and the natural ligands.

**Table 2.** Results with the Chimera program of interaction between AR (2AM9) with Testosterone.

Compound	Affinity (kcal/mol)	RMSD L.b	RMSD u.b	Bridges hydrogen		
				A	L	R
Testosterone	-10	0.0	0.0	2	2	2
Dihydrotestosterone	-10.8	0.0	0.0	3	3	3

A = all, L = ligand, R = receptor.

**Table 3.** Results with the AutoDock Vina program of interaction between AR (2AM9) with Testosterone.

Compounds	Affinity (Kcal/mol)	RMSD L.b	RMSD u.b
Testosterone	-10.0	0.0	0.0
Dihydrotestosterone	-10.8	0.0	0.0

The results showed that the Dihydrotestosterone (DHT) ligand has a higher affinity for the AR compared to testosterone which is the precursor of DHT. This affinity is reported by Pang *et al.* (2021) who mentioned that DHT has a greater action on the receptor and that it is active in the functions in which AR is involved, such as in the transcription of genes for survival and cell growth. ^15^ The validation of the established parameters of the normal androgen receptor binding site, with the use of receptor inhibitors, reflects that the ligand binding site corresponds to the binding site of the natural ligands (testosterone and DHT); however, the drug Enzalutamide and Apalutamide showed high affinity for another receptor site. Also, Chen *et al.* (2019) mentioned that Enzalutamide and Apalutamide are considered second-generation antiandrogens which inhibit the activity of AR during cancer development, whereas first-generation drugs included Bicalutamide, Flutamide and Nilutamide. ^16^ The results obtained were presented in Table 4.

**Table 4.** Affinity of first-generation antiandrogens against PCa to the binding site with AutoDock Vina.

Compounds	Affinity (Kcal/mol)	RMSD L.b	RMSD u.b
Bicalutamide	-8.3	0.00	0.00
Flutamide	-7.7	0.00	0.00
Nilutamide	-8.6	0.00	0.00
Enzalutamide	0.3	0.00	0.00
Apalutamide	0.4	0.00	0.00

The results obtained with Enzalutamide (0.3) and Apalutamide (0.4) showed a low affinity to the common binding site of androgen receptor, so it was analyzed in which area of the receptor these molecules were bound and what is their affinity, under a cube size of 40×40×40 Å ( Table 5).

**Table 5.** Affinity of second-generation antiandrogens against PCa to any site in the wild-type receptor LBD region (2am9).

Compounds	Affinity (Kcal/mol)	RMSD L.b	RMSD u.b
Enzalutamide	-7.6	0.00	0.00
Apalutamide	-7.7	0.00	0.00

## Discussion

**Figure 1.**
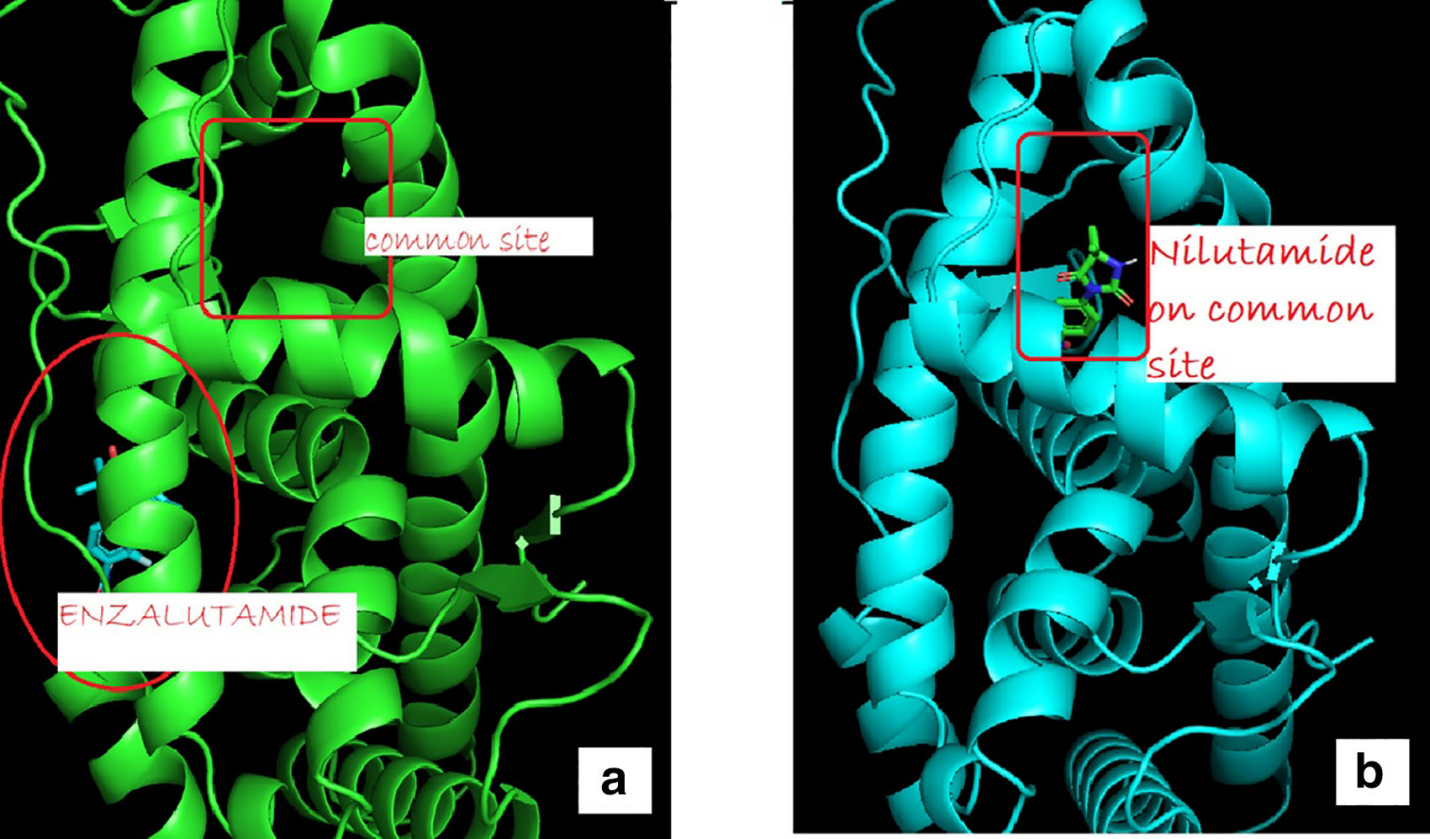
The binding site of androgen receptor inhibitor drugs both first generation (such as nilutamide) and second generation (such as Enzalutamide). (a) showed uncommon androgen receptor and site of enzalutamide binding (b) show wild type androgen receptor bind to common site.

**Figure 2.**
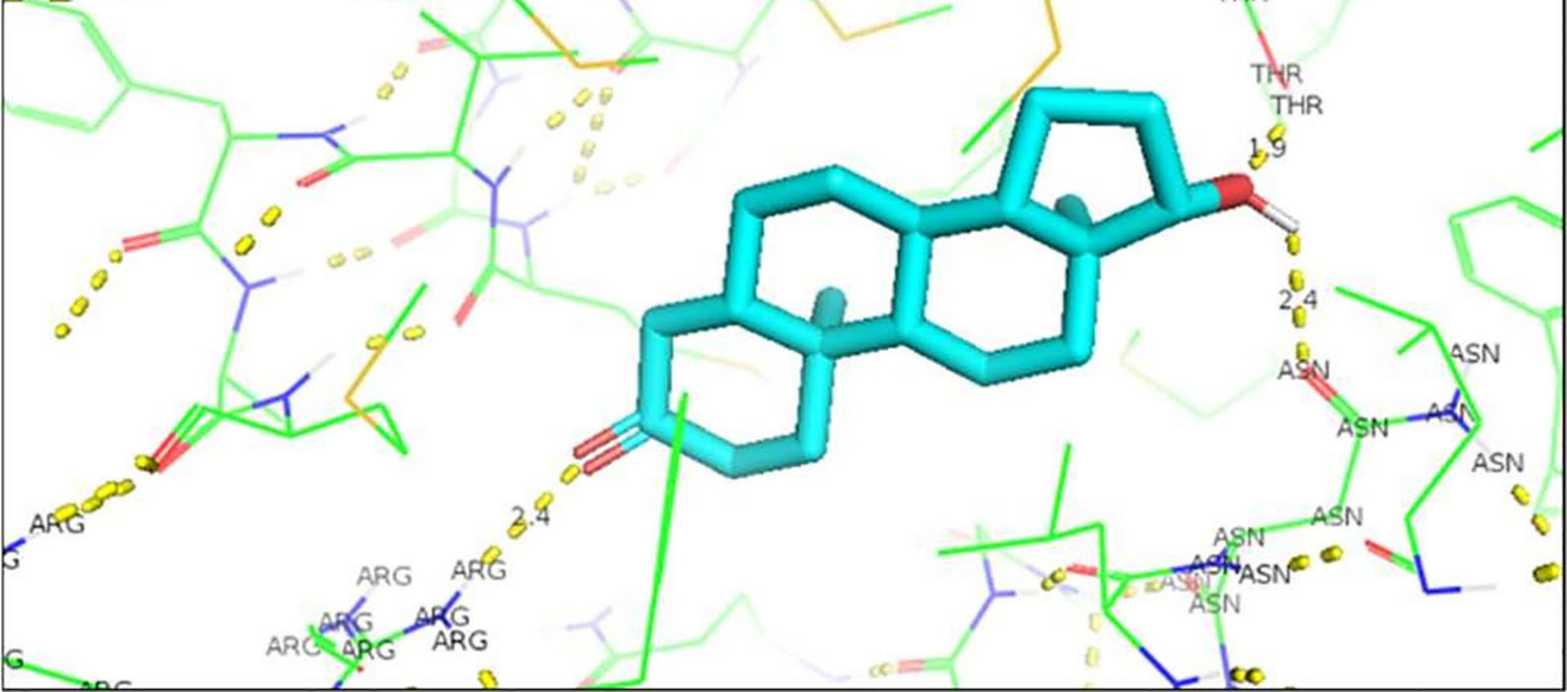
Dihydrotestosterone-wild androgen receptor interaction.

**Figure 3.**
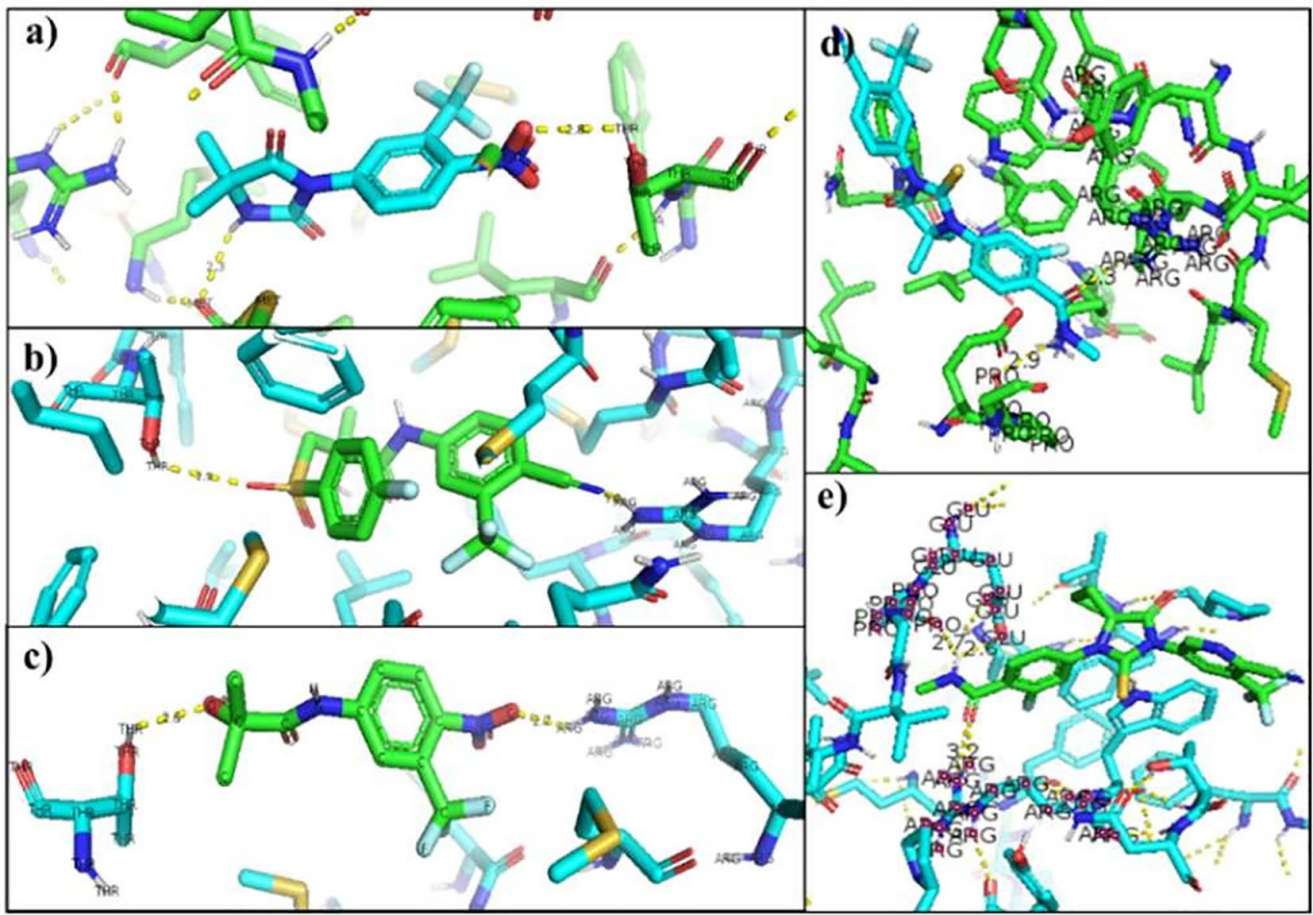
Inhibitor-wild androgen receptor interaction. a) Nilutamide, b) Bicalutamide, c) Flutamide, d) Enzalutamide and e) Apalutamide.

The change in the affinity of Enzalutamide and Apalutamide was due to the interaction of these drugs with another area different from the normal binding site of AR, giving an increase in of 0.3 (Enzalutamide) and 0.4 (Apalutamide) to -7.6 and -7.7, respectively show in Figure 1. To visualize the interactions of the complexes, the PyMOL program was used, with which the amino acid residues that are involved in the interactions between AR with natural ligands were determined, as with inhibitory ligands. For the wild-type AR complex and the natural ligands, it was observed that they are determined by links between the residues threonine 877 with H of Testosterone (2.4 Å) and arginine 752 with O (2.3 Å) ( Figure 1). In the case of the wild AR complex with dihydrotestosterone, the affinity is greater than that observed with Testosterone, said affinity is given by the bonds between the molecules, the residues involved in the formation of said complex are given by arginine 752 with union of 2.4 Å with the terminal nitrogen of DHT, followed by the union between threonine 877 with the opposite oxygen at a distance of 1.9 Å and finally the third interaction occurs between asparagine with the terminal O of DHT, this union is given at a distance of 2.4 Å ( Figure 2). Table 6 showed the interactions between AR and inhibitory ligands, the residues involved are described as the distance between the molecules ( Figure 3).

**Table 6.** Interactions between wild-type androgen receptor with inhibitors.

Receptor			Distance
2AM9	Bicalutamide	Arginine 752 – N	1.8 Å
		Threonine 877 – O	2.9 Å
2AM9	Flutamide	Arginine 752 – N	2.7 Å
		Threonine 877 – O	2.6 Å
2AM9	Nilutamide	Threonine 877 – O	2.3 Å
		Methionine 745-H	2.8 Å
2AM9	Enzalutamide	Arginine 752 – O	2.3 Å
		Glutamine 681 – H	2.9 Å
2AM9	Apalutamide	Arginine 752 – O	2.6 Å
		Glutamine 681 – H	3.2 Å
		Proline 682–H	2.7 Å

The results of the interactions of the receptor (with the T877A mutation) with the ligands analyzed above, the affinity of the natural ligands is low, compared to that analyzed in the wild receptor. The affinity of the antiandrogen Bicalutamide, if it has an affinity of -8.3, the receptor with the T877A mutation decreases to -4.3, however, the affinity towards Flutamide and Nilutamide increased from -7.7 and -8.6 to -8.7 and -9.3, respectively, and second-generation antiandrogens (Enzalutamide and Apalutamide) were stable, as the T877A mutation is not found at the site where these two drugs bind to inhibit the receptor ( Tables 7 and 8). ^16^ It is noted that a drug binds to the receptors somewhat well, and this confirms the studies that led to the adoption of a drug for treatment apalutamide and enzalutamide binding affinity not changed in both mutated and wild type androgenic receptors which provides an idea to use them even in patient with CRPC and this result augmenting the FDA on (9-2021) approval's of apalutamide for treatment non-metastatic castration-resistant prostate cancer.

**Table 7.** Affinity of first-generation antiandrogens against mutated androgenic receptor (T877A) to the binding site with AutoDock Vina.

Compounds	Affinity (Kcal/mol)	RMSD L.b	RMSD u.b
Testosterone	-5.3	1.779	3.212
Dihydrotestosterone	-6.7	1.448	2.897
Bicalutamide	-4.3	0.00	0.00
Flutamide	-8.7	0.00	0.00
Nilutamide	-9.3	0.00	0.00

**Table 8.** Affinity of second-generation antiandrogens against PCa at any site in the LBD region of the mutated receptor (T877A).

Compounds	Affinity (Kcal/mol)	RMSD L.b	RMSD u.b
Enzalutamide	-7.3	0.00	0.00
Apalutamide	-7.3	0.00	0.00

**Figure 4.**
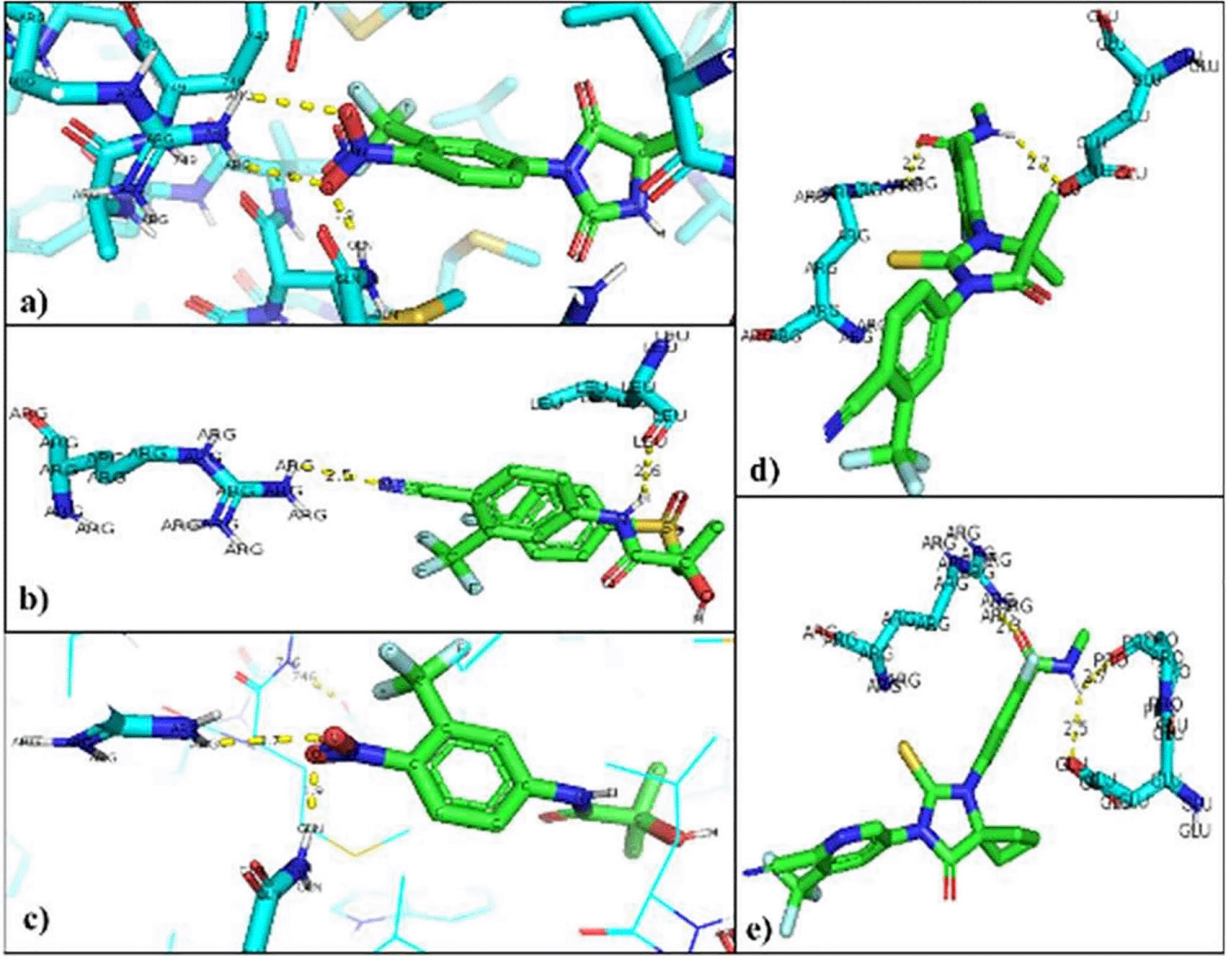
Inhibitor-mutated androgen receptor interaction. a) Nilutamide, b) Bicalutamide, c) Flutamide, d) Enzalutamide and e) Apalutamide.

The interacting residues in each of the mutated AR-ligand complexes were visualized with the PyMOL program. The residues that are modified in the normal interaction of the natural ligands of AR were Threonine 877, so that the union with the natural ligands was between Arginine 752 that binds 9 to O of the Testosterone ligand (2.8 Å), and the Glutamine residue 711 bound to H of the ligand (2.0 Å). In the AR-Dihydrotestosterone complex the amino acids involved are Arginine 752 and Glutamine 711 at a distance of 2.9 Å with O and 2.4 Å with H, respectively ( Figure 4). ^17^^,^^18^

## Conclusion

The preliminary conclusion of this work is that in addition to the normal receptor ligand binding site, there is another site on which the search and identification of drugs can be based. Considering their nature, in this way, a model can be established to search for key sites and potent inhibitors against abnormal androgen receptor function in prostate cancer. In addition, based on computational methodologies, the process of identification and design of therapeutic molecules can be optimized and accelerated. In the same way, the repositioning of drugs will facilitate and increase the efficiency of using existing drugs, which will reduce costs of their implementation by providing a therapeutic alternative in a shorter period of time against prostate cancer.

## Data availability

### Underlying data

Zenodo. Docking result of androgen. DOI: https://doi.org/10.5281/zenodo.5987597 ^20^

This project contains the following underlying data:

- The docking result of androgen and androgenic blocker by using autodock tools and autodock vena

Data are available under the terms of the Creative Commons Attribution 4.0 International license (CC-BY 4.0).
