# *In silico* comparison between the mutated and wild-type androgen receptors and their influence on the selection of optimum androgenic receptor blockers for the treatment of prostate cancer

**DOI:** 10.12688/f1000research.110072.4

**Published:** 2024-01-11

**Authors:** Mohammed J. AL-Zobaidy, Hany Akeel Al-Hussaniy, Zahraa S. Al-tameemi

**Affiliations:** 1 Department of Pharmacology, College of Medicine, University of Baghdad, Baghdad, Iraq; 2 Department of Pharmacy, Bilad Alrafidain University College, Diyala Junction, Baqubah, Diyala, Iraq; 3 Dr Hany Akeel Institute, Iraqi Medical Research Center, Baghdad, Iraq

**Keywords:** androgenic antagonist, anticancer, androgen receptor, molecular docking

## Abstract

**Background**: Prostate cancer is a disease that occurs in men aged more than 50 years. In Iraq, 8.89 men per 100,000 population suffer from prostate cancer, with the incidence being 14,016 cases and mortality being 6,367 cases. Despite advances in treatment against prostate cancer, it can become resistant to drugs. Therefore, the aim of current study was to search and identify binding sites for the repositioning of drugs by computational methods (docking).

**Methods**: Based on the protein structure of the wild androgen receptor, the analysis parameters (22x22x22 on the X, Y, and Z axes) were established.

**Results**: The interactions of the natural ligands with androgen receptor were 10.0 (testosterone) and 10.8 (dihydrotestosterone) while mutated androgen receptor (T877A) had a low affinity with testosterone and dihydrotestosterone (-5.3 and -6.7, respectively). In the interactions of both receptors with the reported inhibitors (antagonists), a decrease with Bicalutamide (-8.3 and -4.3, respectively) and an increase in affinity with Flutamide and Nilutamide (-7.7 and 8.6, wild AR; -8.7 and -9.3 AR T877A) were observed. As for Enzalutamide and Apalutamide (second-generation antagonists), the change was minimal between wild androgen receptor and T877A (-7.6 and -7.7; -7.3 and -7.3, respectively). The change in the affinity of the ligands with androgen receptor and androgen receptor T877A shows how a mutation alters the bonds between these molecules.

**Conclusion**: The identification of key sites and potent inhibitors against abnormal androgen receptor functions will enrich prostate cancer treatments.

## Introduction

Prostate cancer (PCa) is a malignant growth that occurs in men over the age of 50 years and consists of an increase in prostate size due to an increase in the number of cells. In Iraq, this disease has low incidence and mortality according to previous studies, as 7.6% of all types of cancers are prostate cancer. ^1^^,^^2^ In addition, the most likely age for development is between 50 and 74 years. Furthermore, the progression of PCa is related to excess androgen stimulation; however, current treatments include surgery and/or hormones that completely block androgenic receptors. ^3^^,^^4^

When the disease reaches a stage of resistance to treatments, it is called Castration-Resistant Prostate Cancer (CRPC). ^5^ Although new treatment strategies have been developed for CRPC, they are very few and quite inefficient. Many types of CRPC rely on decreasing the activity of the androgen receptor (AR), the signaling pathway for survival. Due to this, the androgen receptor is key to the design of new therapeutic agents. ^5^ To date, more than 600 different mutations have been found in the androgen receptors where the repercussions of these mutations on their (receptors) structure, signaling, and resistance to PCa treatments are analyzed. For this reason, the development of strategies for the identification of effective drugs acting on androgen receptors is of great importance in obtaining new therapeutic agents against PCa. ^6^ This methodology is known as Intensive Structure-Based Virtual Screening (SBVS). ^5^^,^^6^ With the SBVS you can identify new bioactive compounds, make modifications in the structure of molecules, and look for the conformation and optimal position of a ligand with its target molecule to adjust it for the purpose of the drug. ^6^^–^^9^ In addition, the methodology of drug repositioning, which accelerates the process of discovering new uses of existing therapeutic agents, is both cost- and time-effective. Therefore, the use of computational technologies based on protein structure (SBVS) for the design or repositioning of drugs (new uses of existing drugs) is an alternative method that will favor the investigation of new therapeutic agents against CRPC. ^10^

### Castration-Resistant prostate cancer treatment

Despite the cancer being castration-resistant, it may still depend on androgens for growth. Therefore, medications like abiraterone acetate (Zytiga) and enzalutamide (Xtandi) can be effective. These drugs work by either reducing androgen production or preventing androgens from activating their receptors. ^11^ However, enzalutamide failed in some cases and led to resistance. ^11^ There are several research studies attempting to understand the optimal treatment for CRPC and the reasons for this resistance. One such study registered as a clinical trial (NCT02346578) concluded that in patients unresponsive to bicalutamide-combined androgen blockade treatment, enzalutamide demonstrated superior clinical outcomes compared to flutamide. Hence, enzalutamide is recommended over flutamide for these patients.

However, there are no sufficient studies about the reason for this resistance and the binding of this medication to the receptor.

Therefore, the aim of the current study was to find and identify binding sites for both wild-type and mutated androgenic receptors and the best-proposed drug that blocks mutated ones by computational methods (docking); this can give us support or a good explanation of the resistance.

## Methods

The *in-silico* experiments were carried out at the Center for Research in Iraqi Medical and Pharmaceutical Research Center (IMRC). Subsequently, background analysis will be carried out with services provided by protein Data bank-EMBL-EBI (The European Bioinformatics Institute). The Current work is a preliminary trial for the selection of pharmacological molecules with inhibitory potential of the normal and mutated androgen receptor (T877A). ^12^^–^^18^

### Collection of 3D structures of the wild and mutated androgen receptor (T877A)

This first stage consisted of obtaining 3D structures of the wild androgen receptor (AR) and a mutated one (T877A) which is the mutant associated with drug resistance. The 3D molecules were obtained through the database of the protein data bank with access codes 2AM9 (wild) and 2AX6 (mutation).

### Collection of the 3D structures of natural ligands and androgen receptor inhibitors

The structures of the natural ligands of the receptor (Testosterone and Dihydrotestosterone) and its inhibitors (Bicalutamide, Nilutamide, Flutamide, Enzalutamide, and Apalutamide) were obtained from the free database ZINC ( http://zinc15.docking.org). These ligands were selected based on the affinity reported in the database ChEMBL.

### Adequacy of the wild and mutated androgen receptor

The preparation of AR was carried out through the Chimera USFC program, and this preparation consisted of the addition of hydrogen atoms, removal of water molecules from the protein surface, elimination of ligands present, and the determination of charges that integrate each atom of the receiver. In this case, the selected charges were the AM-BCC [AM1 bond charge corrections BCCs]. Subsequently, it was identified and selected based on the basic characteristics of the active androgen receptor site according to PDB [protein data Bank] data.

### Adequacy of the natural ligands and androgen receptor inhibitors

Ligands, both natural and those reported as inhibitors of AR activity, were subjected to adaptations with the Chimera USFC program for molecular interaction assays with the *in-silico* androgen receptor, adding hydrogen atoms and assigning Gastieger charges to mimic the changes that occur within a cell according to their nature. ^14^

### Assays of wild AR interactions with natural ligands and AR inhibitors to establish interaction parameters

The affinity values (Kcal/mol) were calculated using the AutoDock program, the interactions between wild AR and its natural ligands were analyzed, and the contact between the molecules when the distance was less than or equal to 5Å was considered. Based on the interaction of natural ligands (Testosterone and Dihydrotestosterone), the optimal parameters for evaluating receptor interactions were determined. For validation of these parameters, the interactions of the wild androgen receptor with the five inhibitors were reported against the receptor (Bicalutamide, Nilutamide, Flutamide, Enzalutamide, and Apalutamide). ^15^ The same distance (5Å) is considered for interaction between ligands with AR. In addition, within the validation of the joining site, the size assessment of the coupling site was carried out for the structures of the ligands, which was defined by establishing a cube with the dimensions of 22×22×22Å, 15×15×15Å, and 10×10×10Å. This was to experimentally determine the exact critical parameters for interactions of the receptor with the ligands. ^16^

### Assays of interactions of mutated AR (T877A) with natural ligands and AR inhibitors

The interaction analyses between the mutated androgen receptor (T877A) were performed based on the calculations mentioned above, considering the binding site’s size is determined for wild AR with natural ligands and inhibitors (22×22×22Å). This is to determine if mutation changes affinity of the ligands and/or inhibitors to the receptor.

### Identification of the central residues involved in the interaction of wild and mutated AR (T877A) with the ligands

The identification of residues of the AR that interact with the natural ligands and inhibitors, that had the best affinity score in the analyses in AutoDock Vina (Kcal/mol), were visualized using the PyMOL program with which each complex between the receptor and the ligand was observed.

## Results

The first result obtained from the analysis was carried out with the help of the optimal parameters of the AR binding site which were determined with the use of AutoDock Vina and Chimera. The parameters are described below ( Table 1).

**Table 1.** Wild androgen receptor binding site parameters (2AM9).

	X	Y	Z
Center	24.23	4.61	6.129
Size	22	22	22

With the server of AutoDock Vina de Chimera and AutoDock Vina (direct program), the calculation of the theoretical value for the affinity of the coupling of the natural ligand or inhibitors with androgen receptors, both wild and mutated. The following Tables 2 and 3 showed the analyses of affinities existed in the interactions between wild AR with the natural ligands.

**Table 2.** Results for the Chimera program of the interaction between AR (2AM9) and Testosterone.

Compound	Affinity (kcal/mol)	RMSD L.b	RMSD u.b	Bridges hydrogen		
				A	L	R
Testosterone	-10	0.0	0.0	2	2	2
Dihydrotestosterone	-10.8	0.0	0.0	3	3	3

A = all, L = ligand, R = receptor.

**Table 3.** Results for the AutoDock Vina program of the interaction between AR (2AM9) with Testosterone.

Compounds	Affinity (Kcal/mol)	RMSD L.b	RMSD u.b
Testosterone	-10.0	0.0	0.0
Dihydrotestosterone	-10.8	0.0	0.0

The results showed that Dihydrotestosterone (DHT) ligand has a higher affinity for the AR than testosterone, which is the precursor of DHT. Pang *et al.* (2021) reported this affinity and mentioned that DHT has a significant effect on the receptor and is active in the functions in which AR is involved, such as transcription of genes for cell survival and growth. ^17^ The validation of the established parameters of the normal androgen receptor binding site, with the use of receptor inhibitors, reflects that the ligand binding site corresponds to the binding site of the natural ligands (testosterone and DHT); however, the drug Enzalutamide and Apalutamide showed high affinity for another receptor site. Also, Chen *et al.* (2019) mentioned that Enzalutamide and Apalutamide are second-generation antiandrogens that inhibit AR’s activity during cancer development. In contrast, first-generation drugs included Bicalutamide, Flutamide, and Nilutamide. ^18^ The results obtained were presented in Table 4.

**Table 4.** The affinity of first-generation antiandrogens, used in PCa, for the binding site of AR with AutoDock Vina.

Compounds	Affinity (Kcal/mol)	RMSD L.b	RMSD u.b
Bicalutamide	-8.3	0.00	0.00
Flutamide	-7.7	0.00	0.00
Nilutamide	-8.6	0.00	0.00
Enzalutamide	0.3	0.00	0.00
Apalutamide	0.4	0.00	0.00

The results obtained with Enzalutamide (0.3) and Apalutamide (0.4) showed a low affinity for the common binding site of the androgen receptor, so it was analyzed that in which area of the receptor do these molecules bind and what is their affinity under a cube size of 40×40×40Å ( Table 5).

**Table 5.** The affinity of second-generation antiandrogens, used in PCa, for any site in the wild-type receptor LBD region (2am9).

Compounds	Affinity (Kcal/mol)	RMSD L.b	RMSD u.b
Enzalutamide	-7.6	0.00	0.00
Apalutamide	-7.7	0.00	0.00

## Discussion

**Figure 1.**
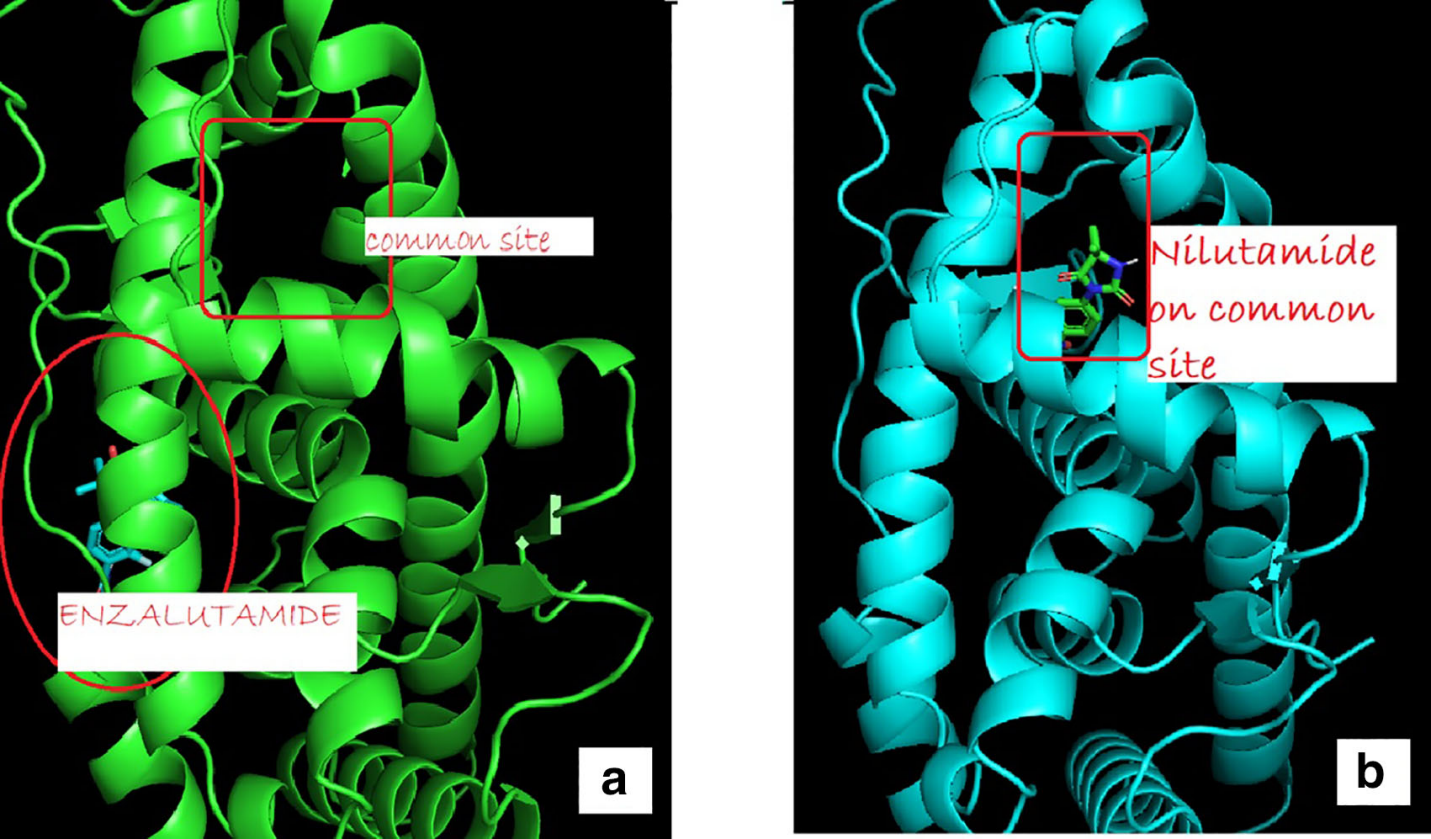
The binding site of androgen receptor inhibitor drugs for both first generation (nilutamide) and second generation (Enzalutamide) drugs. (a) showed uncommon androgen receptors and site of enzalutamide binding (b) showed wild-type androgen receptor binding to a common site.

**Figure 2.**
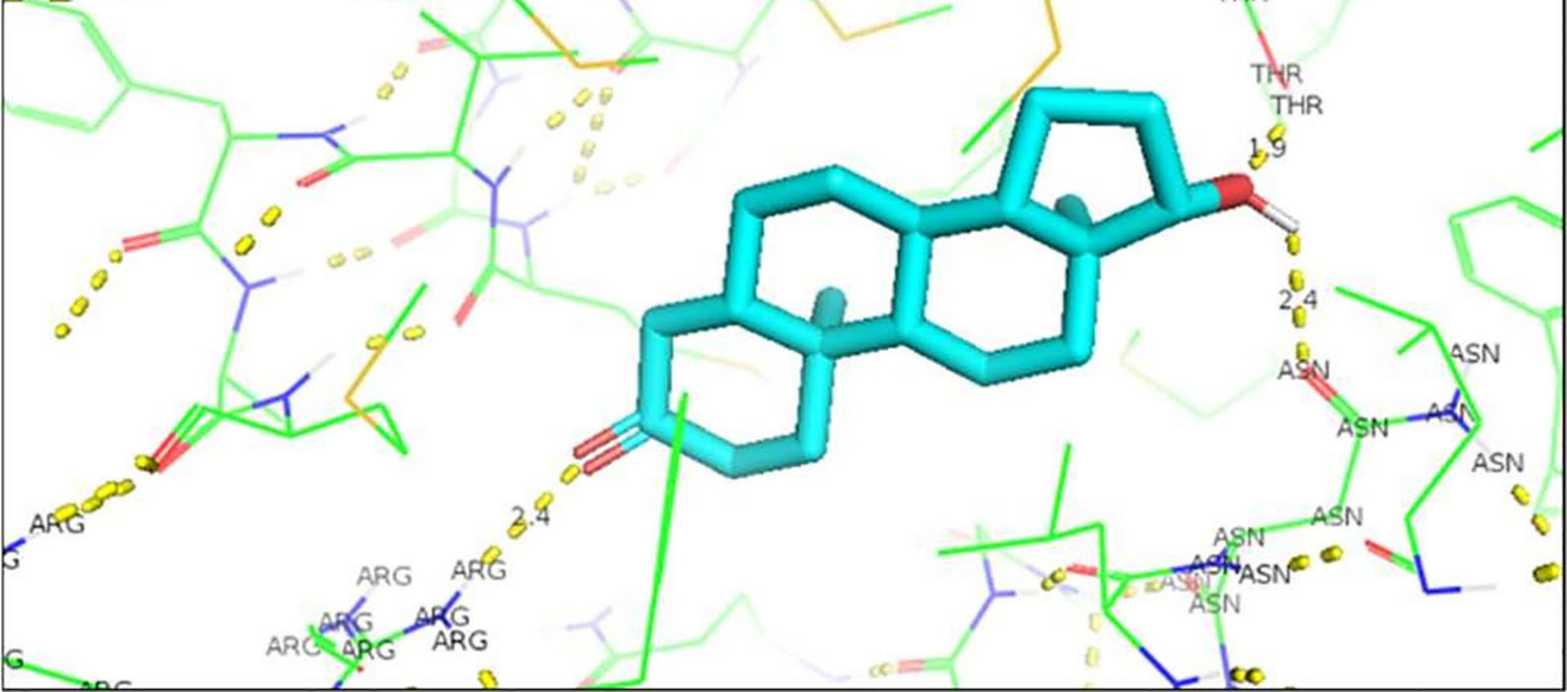
Dihydrotestosterone-wild androgen receptor interaction.

**Figure 3.**
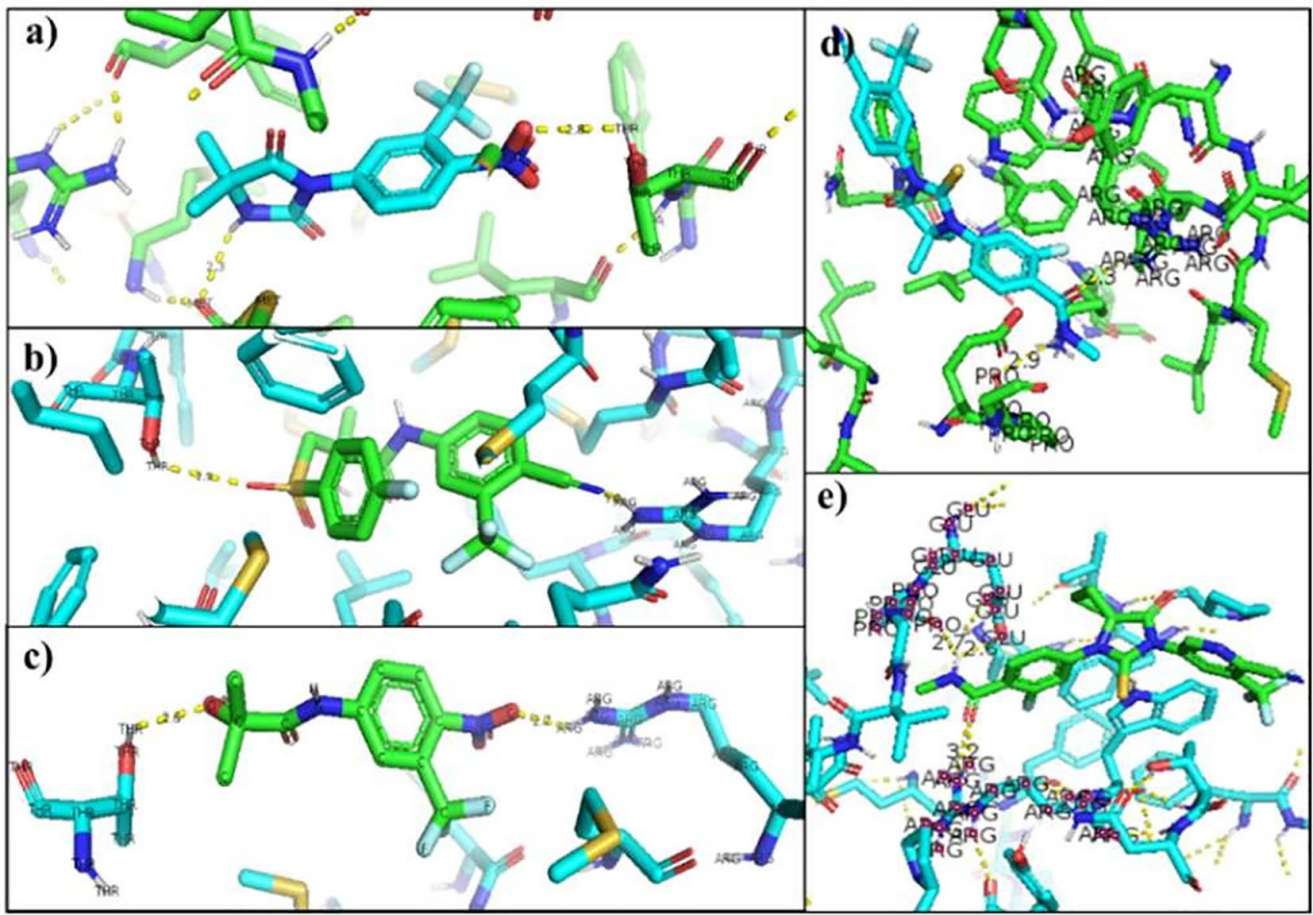
Inhibitor-wild androgen receptor interaction. a) Nilutamide, b) Bicalutamide, c) Flutamide, d) Enzalutamide and e) Apalutamide.

The change in Enzalutamide and Apalutamide was due to the interaction of these drugs with another area different from the normal, expected, binding site of AR, giving an increase of 0.3 (Enzalutamide) and 0.4 (Apalutamide) to -7.6 and -7.7, respectively ( Figure 1). To visualize the interactions of the complexes, the PyMOL program was used, with which the amino acid residue involved in the interactions between AR with natural ligands were determined, as with inhibitory ligands. For the wild-type AR and the natural ligand, testosterone, complex, it was observed that they are determined by links between the residues threonine 877 with H (2.4Å) and arginine 752 with O (2.3Å) ( Figure 1). In the case of the wild-type AR and dihydrotestosterone complex, the affinity is greater than that observed with Testosterone, and the residues involved in the formation of said complex are given by arginine 752 with the terminal nitrogen of DHT (2.4Å), followed by the association between threonine 877 with the opposite oxygen at a distance of 1.9Å, and finally the third interaction occurs between asparagine with the terminal O of DHT, this union is given at a distance of 2.4Å ( Figure 2). Moreover, Table 6 showed the interactions between AR and inhibitory ligands, the residues involved are described as the distance between the molecules ( Figure 3).

**Table 6.** Interactions between wild-type androgen receptors with inhibitors.

Receptor			Distance
2AM9	Bicalutamide	Arginine 752 – N	1.8Å
		Threonine 877 – O	2.9Å
2AM9	Flutamide	Arginine 752 – N	2.7Å
		Threonine 877 – O	2.6Å
2AM9	Nilutamide	Threonine 877 – O	2.3Å
		Methionine 745 – H	2.8Å
2AM9	Enzalutamide	Arginine 752 – O	2.3Å
		Glutamine 681 – H	2.9Å
2AM9	Apalutamide	Arginine 752 – O	2.6Å
		Glutamine 681 – H	3.2Å
		Proline 682 – H	2.7Å

The results of the interactions of the receptor (with the T877A mutation) with the ligands analyzed above showed that the affinity of the natural ligands is low compared to that studied in the wild receptor. The affinity of the antiandrogen Bicalutamide, if it has an affinity of -8.3 for the receptor with the T877A mutation, decreased to -4.3. However, the affinity of Flutamide and Nilutamide increased from -7.7 and -8.6 to -8.7 and -9.3, respectively, and second-generation antiandrogens (Enzalutamide and Apalutamide) were stable, as the T877A mutation is not found at the site where these two drugs bind to inhibit the receptor ( Tables 7 and 8). ^17^ It was noted that a drug binds to the receptors somewhat well, and this confirms the studies that led to the adoption of a drug for treatment. ^18^ On the other hand, apalutamide and enzalutamide binding affinities did not change for both mutated and wild-type androgenic receptors, which provides an idea to use them even in patient with CRPC and this result encouraged the FDA on (9-2021) approvals of apalutamide for treatment of non-metastatic castration-resistant prostate cancer.

**Table 7.** The affinity of first-generation antiandrogens for mutated androgenic receptor (T877A) binding site with AutoDock Vina.

Compounds	Affinity (Kcal/mol)	RMSD L.b	RMSD u.b
Testosterone	-5.3	1.779	3.212
Dihydrotestosterone	-6.7	1.448	2.897
Bicalutamide	-4.3	0.00	0.00
Flutamide	-8.7	0.00	0.00
Nilutamide	-9.3	0.00	0.00

**Table 8.** The affinity of second-generation antiandrogens, used in PCa, at any site in the LBD region of the mutated receptor (T877A).

Compounds	Affinity (Kcal/mol)	RMSD L.b	RMSD u.b
Enzalutamide	-7.3	0.00	0.00
Apalutamide	-7.3	0.00	0.00

**Figure 4.**
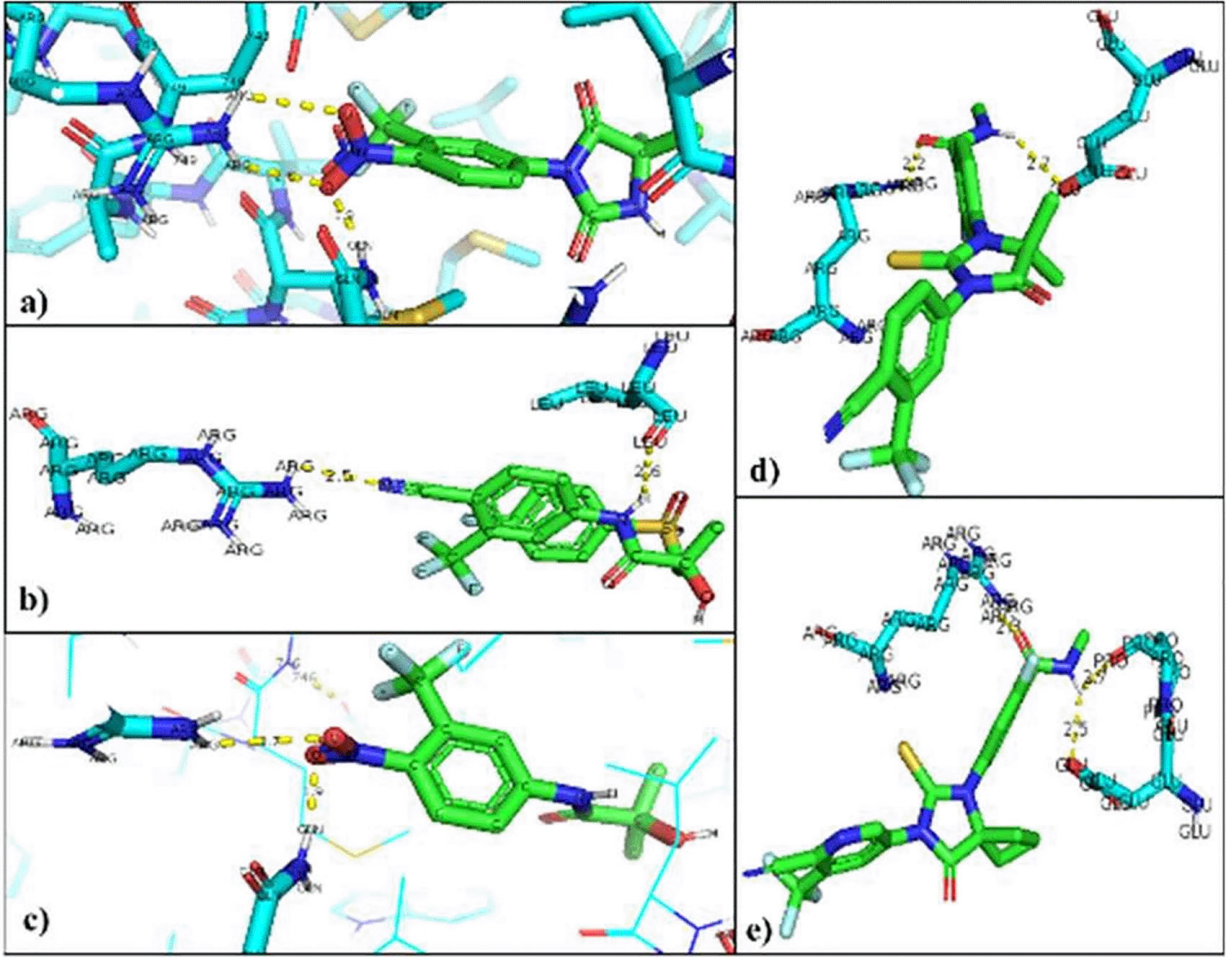
Inhibitor-mutated androgen receptor interaction. a) Nilutamide, b) Bicalutamide, c) Flutamide, d) Enzalutamide and e) Apalutamide.

The interacting residues in each of the mutated AR-ligand complexes were visualized with the PyMOL program. The residues that are modified in the regular interaction of the natural ligands of AR were Threonine 877 so that the union with the natural ligands was between Arginine 752 that binds to O of the Testosterone ligand (2.8Å), and the Glutamine residue 711 attached to H of the ligand (2.0Å). In the AR-Dihydrotestosterone complex, the amino acids involved are Arginine 752 and Glutamine 711 at distances of 2.9Å with O and 2.4Å with H, respectively ( Figure 4). ^19^^,^^20^

## Conclusion

Prostate cancer, specifically the castration-resistant form (CRPC), has continued to be a significant challenge in the medical field. Our study, centering on the identification of binding sites for wild-type and mutated androgenic receptors, provides invaluable insights into potential therapeutic interventions for CRPC. Using *in-silico* methodologies and computational docking techniques, we assessed the binding affinities of known ligands and inhibitors to the androgen receptor (AR), both in its wild and mutated (T877A) forms.

Our findings affirmed that the Dihydrotestosterone (DHT) ligand possesses a higher affinity to the AR compared to testosterone. The different affinities of first and second-generation antiandrogens were elucidated, providing a broader perspective on their effectiveness. Importantly, second-generation antiandrogens, specifically Enzalutamide and Apalutamide, demonstrated alternative binding sites on the AR, which could be an underlying factor contributing to their different therapeutic outcomes in CRPC patients.

Moreover, the observed interactions between the AR and its ligands, facilitated by residues like threonine 877 and arginine 752, offer deeper molecular insights into their binding mechanisms. Such insights are invaluable as they lay the groundwork for designing more targeted therapies in the future.

It is evident from our study that there is a complex interplay of forces governing the interaction between AR and its ligands. This interaction is further complicated by the presence of mutations in the AR, which can potentially alter its binding behavior and drug resistance patterns. Computational methods, such as the ones employed in our study, thus serve as a powerful tool for unraveling these complexities and guiding the future design of therapeutics.

Finally, our study sheds light on the intricate interactions between AR and various ligands, both natural and therapeutic. By understanding these interactions at a molecular level, we open the door to more targeted and effective treatments for CRPC in the future. Further research is essential to translate these findings into tangible clinical outcomes, but the foundation laid by this study is undeniably promising.

## Data availability

### Underlying data

Zenodo. Docking result of androgen. DOI: https://doi.org/10.5281/zenodo.5987597 ^21^

This project contains the following underlying data:

- The docking result of androgen and androgenic blocker by using autodock tools and autodock vena

Data are available under the terms of the Creative Commons Attribution 4.0 International license (CC-BY 4.0).
