# Supplementary material for: In silico comparison between the mutated and wild-type androgen receptors and their influence on the selection of optimum androgenic receptor blockers for the treatment of prostate cancer
Source: F1000Res. 2024 Jan 11;11:516. Originally published 2022 May 12. [Version 4] doi: 10.12688/f1000research.110072.4 (PMC11109556; doi:10.12688/f1000research.110072.4)
Supplement: Peer review history

# Reviewer report: Reviewer response for version 4

**DOI:** 10.5256/f1000research.155650.r278968

**Published:** 2024-05-24

**Authors:** Mahmoud A. Al-Sha'er

**Affiliations:** 1 Faculty of Pharmacy, Zarqa University, Zarqa, Jordan

The authors have answered all requested questions, and I approve the revised manuscript.

Is the work clearly and accurately presented and does it cite the current literature?

Yes

If applicable, is the statistical analysis and its interpretation appropriate?

Yes

Are all the source data underlying the results available to ensure full reproducibility?

Yes

Is the study design appropriate and is the work technically sound?

Yes

Are the conclusions drawn adequately supported by the results?

Yes

Are sufficient details of methods and analysis provided to allow replication by others?

Yes

Reviewer Expertise:

NA

I confirm that I have read this submission and believe that I have an appropriate level of expertise to confirm that it is of an acceptable scientific standard.

# Reviewer report: Reviewer response for version 4

**DOI:** 10.5256/f1000research.155650.r268948

**Published:** 2024-05-13

**Authors:** Ahmed A J Mahmood

**Affiliations:** 1 Pharmacy college, University of Mosul, Mosul, Nineveh Governorate, Iraq

Dear authors

 I read the version 4 of you research paper, it is really a very good work. The idea and the goal were very interesting, the writing style and the languages were excellent. Although there are some points and notes that you need to review in order to accomplish your work. The following are some notes that may help you to do so:

1. In the abstract there is some mistakes in writing the docking score for testosterone and dihydrotestosterone, the (–) is missing.
2. Also, in the abstract you need to minimize mentioning the docking score values and replace them by increasing or decreasing, and only the important value must be mentioned here.
3. I would prefer if you merge the tables of the docking score for the two enzymes in one table to facilitate the comparison of the scores data, although they were very clear in their present form, but as I sed it will be easier to the reader.
4. For more accuracy and to potentiate your results, I would prefer to compare the results of the wild enzyme with results of two mutated enzyme and not only one muted enzyme as you presented, this will accurate your data and your conclusion.
5. And if the previous point can not be achieved, I would prefer to do the MD simulation for selected ligands with the both enzymes pocket, this also would potentiate your results and conclusions.
6. In the conclusion it will be more convenient to propose the best agents that your results made it a promising agent for the treatment of the prostate cancer.

finally, accept my best wishes.

Is the work clearly and accurately presented and does it cite the current literature?

Yes

If applicable, is the statistical analysis and its interpretation appropriate?

Not applicable

Are all the source data underlying the results available to ensure full reproducibility?

Yes

Is the study design appropriate and is the work technically sound?

Yes

Are the conclusions drawn adequately supported by the results?

Yes

Are sufficient details of methods and analysis provided to allow replication by others?

Yes

Reviewer Expertise:

pharmaceutical chemistry and drug design

I confirm that I have read this submission and believe that I have an appropriate level of expertise to confirm that it is of an acceptable scientific standard.

# Reviewer report: Reviewer response for version 4

**DOI:** 10.5256/f1000research.155650.r237280

**Published:** 2024-01-27

**Authors:** Zarrin Basharat

**Affiliations:** 1 Alpha Genomics Private Limited (Ringgold ID: 63596), Islamabad, Pakistan

The authors have improved the manuscript. I recommend acceptance.

Is the work clearly and accurately presented and does it cite the current literature?

No

If applicable, is the statistical analysis and its interpretation appropriate?

Partly

Are all the source data underlying the results available to ensure full reproducibility?

Yes

Is the study design appropriate and is the work technically sound?

Partly

Are the conclusions drawn adequately supported by the results?

Partly

Are sufficient details of methods and analysis provided to allow replication by others?

Yes

Reviewer Expertise:

Drug design, docking simulation, microbiology, bioinformatics, proteomics, genomics

I confirm that I have read this submission and believe that I have an appropriate level of expertise to confirm that it is of an acceptable scientific standard.

# Reviewer report: Reviewer response for version 3

**DOI:** 10.5256/f1000research.139240.r164311

**Published:** 2023-02-24

**Authors:** Zarrin Basharat

**Affiliations:** 1 Alpha Genomics Private Limited (Ringgold ID: 63596), Islamabad, Pakistan

The study aimed to repurpose some drugs against mutated androgen receptor ligand binding domain and could have clinical translation prospects. Authors took publicly available data and used Autodock for docking. The results showed that the mutated receptor had lower affinity towards some ligands ligands while having variable affinity towards others. However, the protein and ligand preparation as well as validation was not rigorous. The docking is a prediction and can be influenced by various factors such as the accuracy of the protein structure, the scoring function used, and the conformational flexibility of the ligand and receptor. Since authors come from LMIC, with limited resources, they could try and validate using energy calculation and dynamics simulation on the least (as access to experimental setup would be limited). Simply docking scores are not enough in most cases and MM-PBSA values etc have been reported to have better results.

 Authors mention in abstract that they look androgen receptor structure, whereas it is only part of it (ligand binding domain that is present with the mentioned ID in PDB). Authors need to be clear about this. Name of software needs to be mentioned and parameter portion edited. Abstract lack of information on docking parameters: The abstract mentions that analysis parameters were established for docking, but it does not provide any details on the specific algorithms or software used. This makes it difficult to assess the reliability and reproducibility of the docking results.

 Authors should also provide a detailed rationale for selecting the ligands (preferably add a table with references that depict use of these ligands in treatment). The scope is somewhat limited, the study only investigated the interactions of a few ligands and inhibitors with the androgen receptor and a specific mutation (T877A). It is unclear whether these findings can be generalized to other ligands, inhibitors, or mutations. Authors could shed some light on this.

 Language needs to be edited at several places for clarity and conveying proper scientific meaning. The heading ‘Adequacy of the wild and mutated androgen receptor ‘ and the text beneath it needs reassessment and editing. ‘subjected to adaptation’?

 Same goes for ‘Assays of wild AR interactions with natural ligands and AR inhibitors to establishing interaction parameters’ heading. Too long and not correct. Natural ligands can act as control for this study. What is L.b and U.b?

 Authors took a ligand binding section of the protein, how can they write in conclusion that ‘….. in addition to the normal receptor ligand binding site, there is another site on which the search and identification of drugs can be based.’. They did not do a site scoring and binding comparison based on this. Several deficiencies need to be addressed in this study before it can be indexable.

Is the work clearly and accurately presented and does it cite the current literature?

No

If applicable, is the statistical analysis and its interpretation appropriate?

Partly

Are all the source data underlying the results available to ensure full reproducibility?

Yes

Is the study design appropriate and is the work technically sound?

Partly

Are the conclusions drawn adequately supported by the results?

Partly

Are sufficient details of methods and analysis provided to allow replication by others?

Yes

Reviewer Expertise:

Drug design, docking simulation, microbiology, bioinformatics, proteomics, genomics

I confirm that I have read this submission and believe that I have an appropriate level of expertise to state that I do not consider it to be of an acceptable scientific standard, for reasons outlined above.

# Response

**Published:** 2024-01-17

**Authors:** الدكتور هاني عقيل

Reviewer note  (The study aimed to repurpose some drugs against mutated androgen receptor ligand binding domain and could have clinical translation prospects. * **Regarding the aim, was improved.**       

 Authors took publicly available data and used Autodock for docking. The authors took a available data (* actually its my current research supplementary data).     

 The results showed that the mutated receptor had lower affinity towards some ligands ligands while having variable affinity towards others.  ( **Yes we made docking on certain receptor that may be linked to increased resistance to androgenic blocker in Iraq )**  

 However, the protein and ligand preparation as well as validation was not rigorous. **Yes that's correct.**  

 The docking is a prediction and can be influenced by various factors such as the accuracy of the protein structure, the scoring function used, and the conformational flexibility of the ligand and receptor. There are several articles support our work regarding the binding     Since authors come from LMIC, with limited resources, they could try and validate using energy calculation and dynamics simulation on the least ( **as access to experimental setup would be limited).** **There is no requirement of experiments on human or animals because all of this medications is already approved**    

 Simply docking scores are not enough in most cases and MM-PBSA values etc have been reported to have better results **.)** **We used 3 types of docking programs** **Autodock vena, pyrx , molecular operation environment** **To ensure the result of docking **   Regarding clinical studies there is several **clinical studies regarding the antiandrogenic medication for treatment of castration resistance prostate cancer and these clinical studies concluded that Enzalutamide is one of the best choice for treatment, our research support there findings , (Enzalutamide and Apalutamide) were stable, and good inhibitors in case of castration resistance prostate cancer**

 Regarding English language 

**We improved it.**

**Finally I hope you accept my article.**

**Best regards,**

**Hany Akeel Al-hussaniy**
